# First person – Amani Hassan

**DOI:** 10.1242/bio.039792

**Published:** 2019-01-15

## Abstract

First Person is a series of interviews with the first authors of a selection of papers published in Biology Open, helping early-career researchers promote themselves alongside their papers. Amani Hassan is first author on ‘The 17β-estradiol induced upregulation of the adhesion G-protein coupled receptor (ADGRG7) is modulated by ESRα and SP1 complex’, published in BiO. Amani is a PhD student in the lab of Dr Florina Moldovan at CHU Sainte-Justine Research Center, Canada, investigating the underlying mechanisms of adolescent idiopathic scoliosis (AIS).


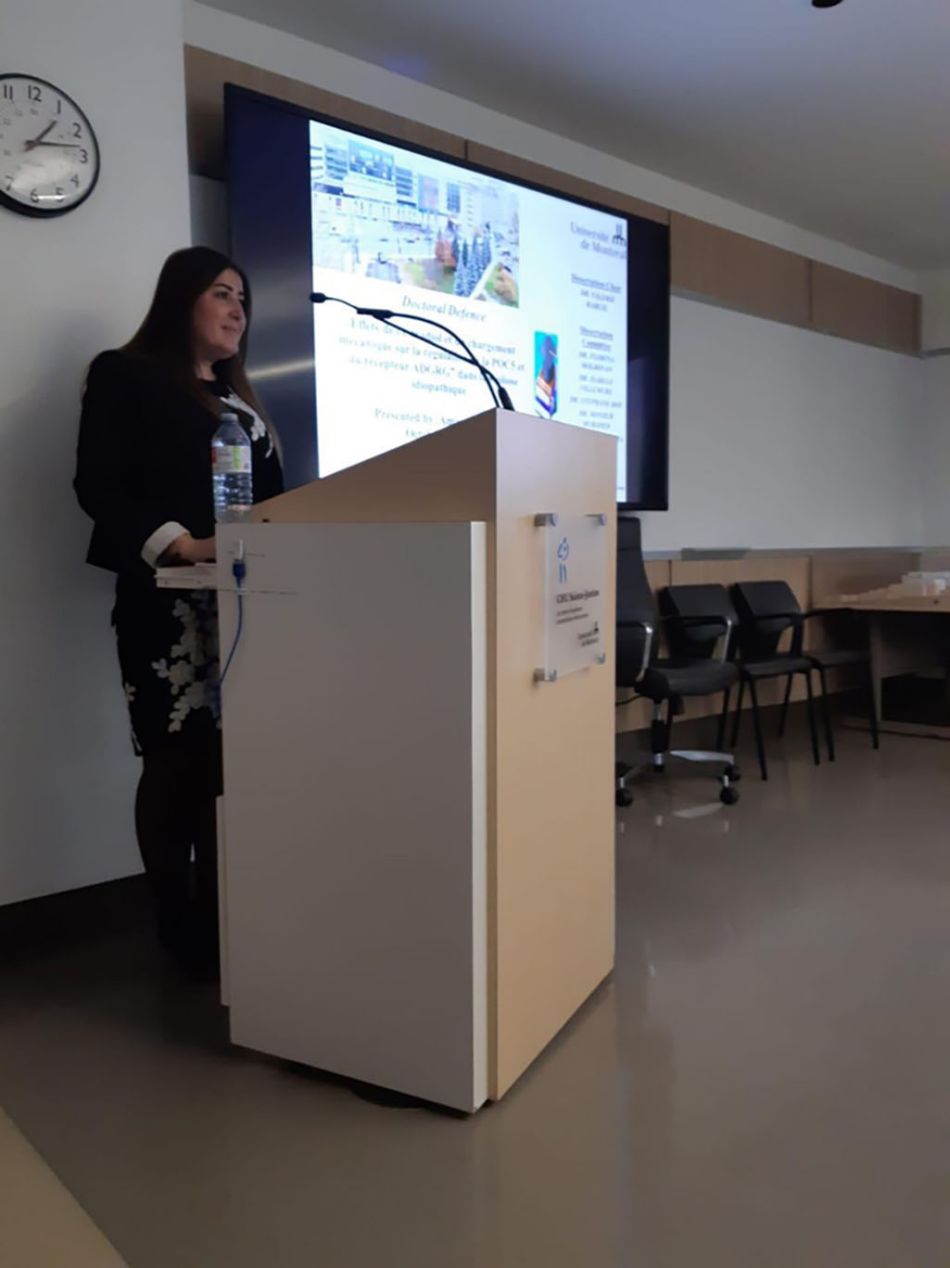


**Amani Hassan**

**What is your scientific background and the general focus of your lab?**

I have a strong background in Biochemistry and Molecular Biology. In our lab, we are interested in studying several factors involved in adolescent idiopathic scoliosis (AIS) development, progression and pathogenesis. The hormonal factor is one of several factors that we believe play a role in AIS. Specifically, evidence suggests that the hormone estrogen (E2) has a role in AIS. Girls are more affected than boys during puberty, a period of life where estrogen has its peak. By applying different molecular biology tools, we study the regulation of genes (ADGRG7 and other genes) by E2.

“Girls are more affected than boys during puberty, a period of life where estrogen has its peak.”

**How would you explain the main findings of your paper to non-scientific family and friends?**

In this work, we found that estrogen, which is a female hormone, plays an important role in the disease AIS. AIS is a bone disease that affects the skeleton causing deformity and rotation. We found that estrogen affects the expression levels of a protein called ADGRG7. Little is known about the protein ADGRG7 and we contribute to the understanding of the role of this protein in AIS in our paper.

**What are the potential implications of these results for your field of research?**

The presented work helps understand the pathogenesis of AIS and the underlying mechanisms of the contribution of estrogen to the disease.

**What has surprised you the most while conducting your research?**

The most surprising issue was that normal cells had a different response to E2 than AIS cells. AIS cells were resistant to E2 treatments.

**What, in your opinion, are some of the greatest achievements in your field and how has this influenced your research?**

Recent work from our lab identified that POC5 and ADGRG7 are both involved in AIS. This work is the basis of my current work and answers the question of why we have selected ADGRG7 for further investigations. Indeed, ADGRG7 has a role in AIS, not as a causative gene, but as contributor gene.

**What changes do you think could improve the professional lives of early-career scientists?**

Good training and experience, exposure to different scientific areas and very good publications would all contribute to improving the lives of early-career scientists.

**What's next for you?**

I would like to pursue my postdoc studies. My future goals are to have my own lab and be a researcher.
